# Tumor suppressor genes and their underlying interactions in paclitaxel resistance in cancer therapy

**DOI:** 10.1186/s12935-016-0290-9

**Published:** 2016-02-20

**Authors:** Jia-Hui Xu, Shi-Lian Hu, Guo-Dong Shen, Gan Shen

**Affiliations:** Department of Geriatrics, Anhui Provincial Hospital affiliated to Anhui Medical University, 17 Lujiang Road, Hefei, 230001 China; Anhui Provincial Key Laboratory of Tumor Immunotherapy and Nutrition Therapy, Hefei, 230001 China

**Keywords:** Tumor suppressor genes, Paclitaxel resistance, GeneMANIA, Molecular functions

## Abstract

**Objectives:**

Paclitaxel (PTX) is frequently used in the clinical treatment of solid tumors. But the PTX-resistance is a great obstacle in cancer treatment. Exploration of the mechanisms of drug resistance suggests that tumor suppressor genes (TSGs) play a key role in the response of chemotherapeutic drugs. TSGs, a set of genes that are often inactivated in cancers, can regulate various biological processes. In this study, an overview of the contribution of TSGs to PTX resistance and their underlying relationship in cancers are reported by using GeneMANIA, a web-based tool for gene/protein function prediction.

**Methods:**

Using PubMed online database and Google web site, the terms “paclitaxel resistance” or “taxol resistance” or “drug resistance” or “chemotherapy resistance”, and “cancer” or “carcinoma”, and “tumor suppressor genes” or “TSGs” or “negative regulated protein” or “antioncogenes” were searched and analyzed. GeneMANIA data base was used to predict gene/protein interactions and functions.

**Results:**

We identified 22 TSGs involved in PTX resistance, including *BRCA1*, *TP53*, *PTEN*, *APC*, *CDKN1A*, *CDKN2A*, *HIN*-*1*, *RASSF1*, *YAP*, *ING4*, *PLK2*, *FBW7*, *BLU*, *LZTS1*, *REST*, *FADD*, *PDCD4*, *TGFBI*, *ING1*, *Bax*, *PinX1* and *hEx*. The TSGs were found to have direct and indirect relationships with each other, and thus they could contribute to PTX resistance as a group. The varied expression status and regulation function of the TSGs on cell cycle in different cancers might play an important role in PTX resistance.

**Conclusion:**

A further understanding of the roles of tumor suppressor genes in drug resistance is an important step to overcome chemotherapy tolerance. Tumor suppressor gene therapy targets the altered genes and signaling pathways and can be a new strategy to reverse chemotherapy resistance.

## Background

Currently, chemotherapy is the main cancer treatment modality, among which paclitaxel (PTX) is a type of cytotoxic agent and widely used in the first line treatment of lung, ovarian, breast, renal cancers and Kaposi’s sarcoma [1–5]. PTX differs from conventional anti-cancer drugs because it does not affect the DNA or RNA synthesis of tumor cells or cause DNA damage, but interferes with tubulin to stabilize microtubule composition and normal spindle assembly and cell division resulting in cancer cell death [6].

The clinical use of PTX leads to variable responses in different individuals, and the mechanisms of PTX resistance have not been fully elucidated. Some reports suggested that tumor suppressor genes (TSGs) should be important mediators of drug sensitivity [7–9]. Normally, these TSGs prevent abnormal cells from surviving. However, when the genes are inactivated or reduce expression, the abnormal cells grow uncontrollably, which may lead to cancer formation [10].

In this study, by the analysis of published reports and GeneMANIA network, we reviewed 21 TSGs and 1 putative TSG that contributed to PTX resistance in cancer and provided an overview of the relationship of TSGs with PTX resistance.

### Overall information on the 22 genes related to PTX resistance in cancer

To comprehensively collect all of the TSGs related to PTX resistance, we searched the PubMed online database and google web site, followed by an advanced search using the terms “paclitaxel response” or “paclitaxel sensitive” and “drug resistance” or “chemotherapy resistance,” and “cancer” or “carcinoma,” and “tumor suppressor genes” or “negative regulated protein” or “antioncogene” This search identified 22 TSGs including breast cancer 1 (*BRCA1*), tumor protein p53 (*TP53*), phosphatase and tension homolog (*PTEN*), adenomatous polyposis coli (*APC*), cyclin-dependent kinase inhibitor 1A (*CDKN1A*), cyclin-dependent kinase inhibitor 2A (*CDKN2A*), high in normal-1 (*HIN*-*1*), ras association domain-containing protein 1 (*RASSF1*), yes-associated protein 1 (YAP), inhibitor of growth 4 (*ING4*), polo-like kinase 2 (*PLK2*), f-box and WD repeat domain containing 7 (*FBW7*), zinc finger MYND type containing 10 (*BLU*), leucine zipper tumor suppressor 1 (*LZTS1*), re-1 silencing transcription factor (*REST*), fas-associated death domain protein (*FADD*), programmed cell death 4 (*PDCD4*), transforming growth factor-β-induced (*TGFBI*), inhibitor of growth 1 (*ING1*), bcl-2-associated X protein (*Bax*), PIN2/TRF1 interacting telomerase inhibitor 1 (*PinX1*) and one putative tumor suppressor gene, FERM domain-containing protein 6 (*hEx*), which contributed to PTX-resistance in cancer. The status, regulation manner, pathway and cancer type involved in PTX-resistance have been summarized, as shown in Table 1.

**Table 1:** General overview of the 22 TSGs that contribute to PTX-resistance

TSG abbreviation	Full name of the TSGs	Status	Regulation manner	Pathway associated with resistance	Type of cancer
*BRCA1*	Breast cancer 1	Mutation [12, 17] Protein/mRNA level [13, 14]	Spindle-assembly checkpoint [15] Microtubule dynamic [16] MEKK3 activity [17]	Apoptosis [13, 16] JNK/SAPK and p38/MAPK pathway [17]	Ovarian cancer [13] HNSCC [14] Breast cancer [15, 17] NSCLC [16]
*TP53*	Tumor protein p53	Mutation [26]	G1 phase arrest [22] Apoptosis [24]	Apoptosis [24]	NSCLC [22] Ovarian cancer [24, 26]
*PTEN*	Phosphatase and tension homolog	Protein level [34, 35]	Cyclin B1 activity [34] MiR-22 [35]	PI3 K/AKT pathway [34, 35]	ESCC [34] Colon cancer [35]
*APC*	Adenomatous polyposis coli	Mutation [38]	MDR1 [38] miR-135a [40]	Cell cycle [40], Cell adhesion [41]	Breast cancer [38] NSCLC [40]
*p21/CDKN1A*	Cyclin-dependent kinase inhibitor 1A	Protein level [48]	Cell cycle [48]	Cell cycle, Apoptosis [48]	Melanoma [48]
*p16/CDKN2A*	Cyclin-dependent kinase inhibitor 2A	Protein level [49]	Cell cycle [49]	Cell cycle [49]	Triple-negative breast cancer [49]
*FRMD6/hEx*	FERM domain-containing protein 6	Protein level [8]	Cell cycle [8]	Cell cycle [8]	Breast cancer [8]
*RASSF1*	Ras association domain-containing protein 1	Methylation [54]	Cell growth [53]	Cell cycle [53]	Ovarian cancer [53]
*YAP*	Yes-associated protein 1	deletion [55]	Cell cycle [55]	Cell cycle [55]	Breast cancer [55]
*ING4*	Inhibitor of growth 4	Protein level [56]	Bcl-2/Bax ratio [56]	Apoptosis, Cell cycle [56]	Lung cancer [51]
*BAX*	BCL2-associated X protein	mRNA level [57]	Bcl-2/Bax ratio [57]	Apoptosis [57]	Breast cancer [57]
*HIN*-*1/SCGB3A1*	High in normal-1	Methylation [9]	Apoptosis [9]	PI3K/AKT pathway [9]	Ovarian cancer [9]
*PLK2*	Polo-like kinase 2	Methylation [58]	G2/M phase checkpoint [58]	Cell cycle, apoptosis [58]	Ovarian cancer [58]
*LZTS1/FEZ1*	Leucine zipper tumor suppressor 1	Protein/mRNA level [59, 60]	Cell cycle [59, 60]	Cell cycle [59, 60]	Ovarian cancer [59] Breast cancer [60]
*FBXW7/FBW7*	F-box and WD repeat domain containing 7	Mutation [62]	Ubiquitination [62]	Ubiquitination [62]	Ovarian cancer [62]
*ZMYND10/BLU*	zinc finger MYND type containing 10	Methylation [63]	Bcl-2/Bax ratio [63, 64]	Apoptosis [63], PI3 K/Akt pathway [59, 64]	Ovarian cancer [63, 64]
*TGFBI*	Transforming growth factor-β-induced	mRNA/protein level [66, 67]	β3 integrin [66, 67]	Apoptosis [66, 67]	NSCLC [66] Ovarian cancer [67]
*REST*	RE-1 silencing transcription factor	Protein level [70]	TUBB3[70]	PI3K/AKT pathway [70]	Ovarian cancer [70]
*FADD*	Fas-associated death domain protein	Phosphorylation [72, 73]	Apoptosis [71] Cell cycle [72]	JNK/SAPK pathway [72]	Cervical carcinoma [71, 73] Prostate cancer [72]
*PDCD4*	Programmed cell death 4	Protein/mRNA level [74, 75]	Mir-182 [74]	Cell growth [74] Cell cycle [75]	Ovarian cancer [74] Cervical carcinoma [75]
*ING1*	Inhibitor of growth 1	Protein level [76]	Apoptosis [76]	p53-dependent pathway [76]	Osteosarcoma [76]
*PinX1*	PIN2/TRF1 interacting telomerase inhibitor 1	Protein level [77]	Spindle-assembly checkpoint [77]	Cell cycle [77]	Cervical carcinoma [77]

### *BRCA1*

Tumor suppressor *BRCA1* is involved in several cellular functions including DNA damage repair, cell cycle checkpoint activation and transcription [11]. Several preclinical studies indicated that *BRCA1* might be an important determinant of response to PTX-based chemotherapy. It was shown that reconstitution of exogenous BRCA1 in the *BRCA1*-mutant HCC1937 breast cancer cell line resulted in enhanced sensitivity to PTX [12]. In accordance, low *BRCA1* mRNA expression in ovarian cancer cell lines resulted in decreased and increased apoptotic response to PTX and platinum respectively and PTX-sensitive human brain and neck squamous cell carcinoma (HNSCC) with acquired cisplatin resistance had high expression of BRCA1 [13, 14]. In order to investigate the underlying PTX-resistance mechanisms conferred by loss of *BRCA1*, Chabalier et al. reduced BRCA1 protein levels by using small interfering RNA (siRNA) in MCF7 breast cancer cells resulted in PTX resistance through premature inactivation of spindle checkpoint [15]. Sung et al. found that *BRCA1* knockdown conferred A549 cells resistance to PTX and sensitivity to cisplatin through improving microtubule dynamics which prevented the formation of stable microtubule for caspase-8 accumulation of PTX induced apoptosis [16]. A further study suggested that *BRCA1* might represent an important mediator of the PTX stress-response dependent c-Jun N-terminal kinase/stress-activated protein kinase (JNK/SAPK) or p38/mitogen-activated protein kinase (p38/MAPK) pathway [17]. Taken together, these studies provided evidence that *BRCA1* mutation or reduced expression could predict the response to PTX–based chemotherapy. *BRCA1* deficiency led to increased microtubule dynamics, impaired cell cycle checkpoint and signaling pathway which rendered less sensitivity to PTX-induced apoptosis. Here we consider that *BRCA1* may become a molecular marker to predict the PTX resistance.

### *TP53*

*TP53* is one of the earliest detected tumor suppressor genes and the most frequently mutated gene in carcinoma. More than half of the *TP53* mutations found in cancers lead to loss of function. Functional p53 participates in various cellular processes including cell cycle progression, cell motility, aging, apoptosis, genetic instability, DNA repair, anti-angiogenesis and cell metabolism [18]. *TP53* gene mutation status has recently been shown to be correlated to PTX-based therapy and prognosis [19–21]. It was also found that an augmented concentration of intracellular p53 protein sensitized three non-small-cell lung carcinoma (NSCLC) cell lines to PTX [22]. p53 upregulated modulator of apoptosis (PUMA) is an important regulator of apoptosis and is involved in drug resistance [23]. It was demonstrated that PUMA was downregulated in PTX-resistant ovarian cell line SKOV3/PTX, and delivery of p53 into SKOV3/PTX could upregulated the expression of PUMA and restored the apoptotic response to PTX [24]. *TP53* hot spot mutation (*TP53*-m273) increased multidrug resistance protein 1 (MDR1, regulating efflux of PTX and doxorubicin) expression and resistance to PTX [25, 26]. In addition, studies suggested that some regulatory factors depended on p53 related pathway may mediated PTX resistance. For example, the up-regulation of inhibitor of apoptosis-stimulating protein of p53 (iASPP), a p53 suppression factor, has been found to affect PTX sensitivity in ovarian cancer by inhibiting both mitotic catastrophe and apoptosis [27]. Astrin, a protein localized with mitotic spindles at M phase, silencing of astrin triggered a p53-dependent apoptotic pathway and induced Hela cells sensitive to PTX [28].

### *PTEN*

*PTEN*, is a negative regulator of the phosphatidylinositol 3-kinase/protein kinase B (PI3k/Akt) signaling pathway. Its dysfunction mutation results in reduced dephosphorylation of phosphatidylinositol 3, 4, 5-triphosphate (PIP3), further increasing cell survival, cell migration, cell size and cell proliferation [29]. Recently, reports mainly concentrate on the role of *PTEN* in the response of human cancer cells to anti-cancer drugs and in multiple drug resistance (MDR) reversion [30–33]. Several reports showed that *PTEN* was involved in PTX resistance. Cyclin B1 plays a key role in G2/M transition. Ou et al. detected suppressing of cyclin B1 protein sensitized esophageal squamous cell carcinoma (ESCC) cells to PTX-induced apoptosis through the PTEN/PI3 k pathway [34]. Overexpression of microRNA 22 (miR-22) reversed PTX-induced cytotoxicity and this function was mediated by the regulation of PTEN levels in *TP53* negative colon cell line [35]. Although *PTEN* is not the primary target of PTX resistance, evidences showed that its regulator can be an important target, such as suppression of cyclin B1, miR-22 or combining with inhibitor of Akt could be an attractive strategy for PTX therapy.

### APC

Tumor suppressor gene *APC* is most commonly mutated and deleted in colorectal cancers, as well as many other epithelial cancers like breast, gastric and lung cancer. The best-known function of the APC protein is the regulation of the Wnt signaling cascade through down-regulation of β-catenin can modulate cell cycle progression, however, APC has many Wnt independent roles, such as microtubule dynamic, cytoskeletal organization and cell adhesion [36, 37]. Since PTX is to interfere with microtubule protein stability, the interaction between *APC* and PTX has been explored. Monica et al. showed loss of *APC* in breast cancer cells from mouse mammary tumor virus promoter-polyoma middle T-antigen (MMTV-PyMT) mouse lead to increased expression of MDR1 after treatment with cisplatin and PTX [38]. It has been demonstrated that *APC* expression is regulated by a microRNA 135a (miR-135a) [39]. So it is not surprising that miR-135a is shown to be involved in PTX resistance by downregulation of APC [40]. Moreover, Ling et al. found *APC*-deficient cancer cells defect in mitotic spindle checkpoint and in cell–cell adhesion and were more resistant to PTX [41, 42]. Consequently, *APC* deficiency impairs the PTX sensitivity of cancer cells by interfering with the mitotic spindle checkpoint and decreasing apoptosis.

### CKIs

Loss of cell cycle control promotes tumorigenesis, key regulators of the cell cycle are a family of serine/threonine kinases: cyclin-dependent kinases (CDKs). CDKs act at different stages of the cell cycle and are responsible for the transition from one cell cycle phase to the next [43]. Endogenous cyclin-dependent kinase inhibitors (CKIs) are negative regulators of CDKs [44]. There are two families of CKIs: the INK4 families, consisted of p16, p15, p18 and p19 which can inhibit the complex of cyclin dependent kinase 4/6 (CDK4/6) and cyclin complex activities. And the CIP/KIP families include p21, p27 and p57, regulate border CDKs [45]. Recently, evidences have showed CKIs family members involved in PTX resistance in human cancers. p21, is required to maintain the G2 arrest after DNA damage [46], the level of p21 expression has been known to play an important role in determining sensitivity of tumor cells to PTX [47], and a remarkable induction of p21 in A375P cells after treatment of PTX and apoptosis induction after mitotic arrest with PTX. However, PTX lightly increased the levels of p21 in A375P/Mdr cells, which exhibited strong resistance to PTX [48]. p16, mainly inhibits CDK4 activity, the loss of p16 expression reduced the response of breast cancer cells to PTX by conferring cancer stem cell properties and the tumorsphere formation was not significantly enhanced [49], those results indicated that CKIs affect PTX efficacy mainly through the cell cycle regulation.

### Hippo signaling pathway

The Hippo signaling pathway, which regulates cell proliferation and apoptosis, is a highly conserved signaling pathway first discovered in Drosophila cells. It also exists in mammals and controls organ size, cell proliferation and apoptosis. The main function of Hippo signaling pathway is to phosphorylate transcriptional co-activator PDZ-binding motif (TAZ) and YAP, preventing them from entering the nucleus and promoting gene transcription which induces cell proliferation, metastasis and invasion [50]. Recent discoveries have identified the Hippo signaling pathway as a new target for cancer chemotherapy resistance [51]. For example, *hEx*, one of the Hippo upstream signal input factors, a putative tumor suppressor gene, overexpression of *hEx* dramatically inhibited breast cancer cell proliferation and sensitivity to PTX [8]. RASSF1A, a member of the *RASSF1* family, is a downstream regulator of Hippo, there are approximately 50 % of ovarian tumors harbor hypermethylation of *RASSF1* [52], investigations shown that overexpression of RASSFIA could increase stabilization of microtubules then restore PTX sensitivity [53]. *YAP*, a nuclear effector of Hippo, has been shown exist in many pathways except in Hippo, it is critical for DNA damage in breast cancer cells as well as in certain types of neuronal apoptosis [54]. It acted as a tumor suppressor in breast cancer and its silencing could induce normal breast epithelia more resistance to PTX effect on cell cycle but not apoptosis [55].

### Other TSGs

In addition to the TSGs mentioned above, abnormity of *ING4*, *Bax*, *HIN*-*1*, *PLK2*, *FBW7*, *LZTS1*, *BLU*, *TGFBI*, *REST*, *FADD*, *PDCD4*, *ING1* and *PinX1* have also been found to mediate PTX resistance in some experiments. The protein level of *ING4* was sharply decreased in PTX-resistance lung cancer cells. In contrast, overexpression of ING4 protein could induce apoptosis and G2/M arrest by decreasing B-cell CLL/lymphoma 2 (Bcl-2)/Bax ratio then reversed PTX-resistance [56]. *Bax* is a proapoptotic *Bcl*-*2* family member that plays a key role in induction of mitochondrial dependent apoptosis. Study found there was an increase of Bcl-2/Bax ratio in PTX-resistant breast cancer cell lines, high ratio reduced the PTX-induced apoptosis in breast cancer and ovarian cancer cells [57]. Hypermethylation downregulated the expression of *HIN*-*1* and weakened the sensitivity to PTX through the PI3k/Akt pathway [9]. Hypermethylation of *PLK2* reduced ovarian cancer cells sensitivity to PTX, accompanied by reduced G2-M arrest and apoptosis [58]. Low protein expression of LZTS1 showed little response in patients who received PTX-based chemotherapy in ovarian carcinoma and breast cancer patients, it was a worse prognosis patients outcome [59, 60], previous study by generating *LZTS1* knockout mice, detected accelerate mitotic progression resistance to PTX-induced M phase arrest by decreasing CDK1 activity [61], indicated cell cycle distribution may be involved in the above two human cancer. Ovarian and colon cancer cells which harbored mutant *FBW7* were more resistant to PTX, functional FBW7 is required to degrade myeloid cell leukemia 1 expression by a ubiquitin ligase SKP1–cullin-1–F-box complex that contains FBW7 [62]. Ovarian cancer patients with methylated *BLU* had significantly shorter progression free survival, in vitro, *BLU* could decrease the Bcl-2/Bax ratio in ovarian cancer cells when encountered with PTX [63, 64]. *TGFBI* acts as a tumor suppressor in lung cancer [65]. Irigoyen et al. identified a strong association between elevated *TGFBI* expression and the response to chemotherapy, *TGFBI* mediated the susceptibility of NSCLC cells to PTX and this may be the result of direct TGFBI induction of cell apoptosis through the binding of its proteolytic fragments to the β3 integrin, the same phenomenon was proven in ovarian cancer [66, 67]. REST directly regulates Akt2, loss of REST leads to a de-regulation of Akt phosphorylation [68]. Tubulin beta 3 class III (TUBB3) was a biomarker of the resistance of chemotherapies [69]. Gao et al. found REST might suppress the expression of TUBB3 to sensitize ovarian cancer cells to PTX by activating the PI3k/Akt pathway [70]. Phosphorylation of FADD affected both upstream and downstream of the JNK/SAPK pathway, which was critical for sensitivity to PTX-induced apoptosis [71–73], and PDCD4 mediated PTX sensitivity through interacting mitotic exit regulation axis, upregulation of microRNA 182 (miR-182) accelerated cell cycle process and enhanced chemo-resistance of ovarian cancer cells to PTX through negatively regulating PDCD4 [74, 75]. P33^ING1^, one of the *ING1* gene products, could enhance PTX-induced apoptosis in human osteosarcoma U2OS cells by p53-dependent pathway and its target genes p21 and Bax were increased [76]. In cervical squamous cell carcinomas, the expression of PinX1 in patients was significantly associated with the response of the combination of PTX and cisplatin chemotherapy. In vitro, knockdown of PinX1 could dramatically enhance PTX effects, whereas the augment of PinX1 levels substantially enhanced the G2 phase cells through influencing spindle assembly checkpoint [77].

Summarily, the pre-transcriptional (epigenetic/genetic), transcriptional and post-transcriptional changes of TSGs contribute to PTX resistance in cancer, which may lead to new treatment methods to overcome drug resistance. Actually, the reversion of epigenetic changes of DNA and gene transfer skills (gene therapy) has already proved to be effective in reversing PTX resistance. For example, 5-aza-2-deoxycytidine (a demethylation agent) reversed the sensitivity to PTX treatment in breast cancer and ovarian cancer cells [9, 78]. Exogenous increased levels of p53 significantly improved the sensitivity of PTX providing a basis for gene therapy [22]. For another example, the CDK4/6 inhibitor was shown to downregulate p16/cyclin D1/CDK4/(retinoblastoma protein)Rb signaling pathway and enhance the cytotoxicity of PTX for *KRAS* mutation-positive lung adenocarcinoma cells [79]. These results encouraged further studies on TSGs associated with PTX resistance in cancers. Moreover, drug resistance was rarely induced by single gene, it was almost caused by two or more genes. For example, tumor suppressor p33^ING1^ markedly increased PTX-induced growth inhibition and apoptosis in *TP53*-wild cells, but not in *TP53*-mutant cells [76]. Twenty two genes were involved in the regulation of PTX resistance in cancers through certain pathways, particularly through cell cycle and apoptosis (Table 1).

### The interaction network of the 22 TSGs

**Fig. 1:**
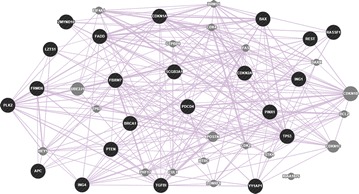
The co-expression network of the 22 TSGs based on GeneMANIA. Co-expression: two genes are linked if their expression levels are similar across conditions in a gene expression study

**Fig. 2:**
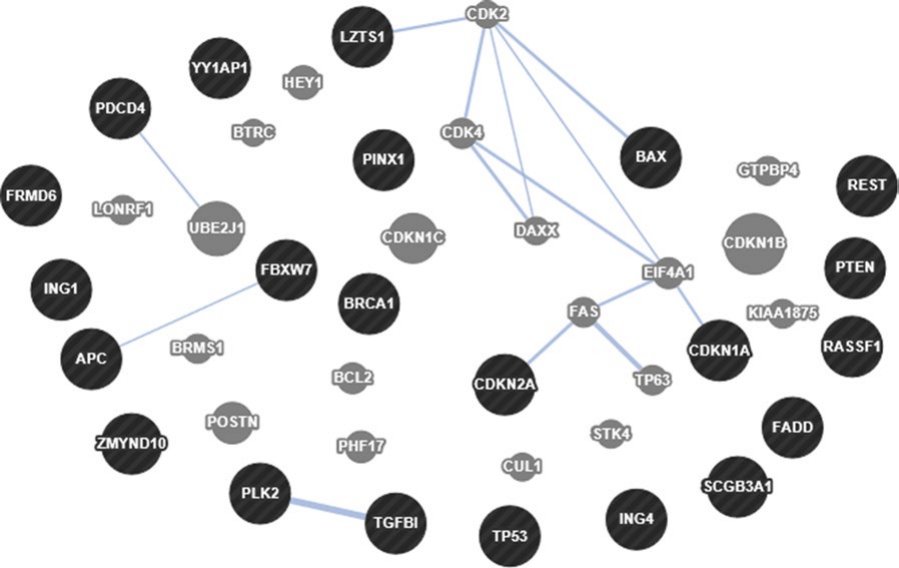
The co-localization network of the 22 TSGs based on GeneMANIA. Co-localization: two genes are linked if they are both expressed in the same tissue or if their gene products are both identified in the same cellular location

**Fig. 3:**
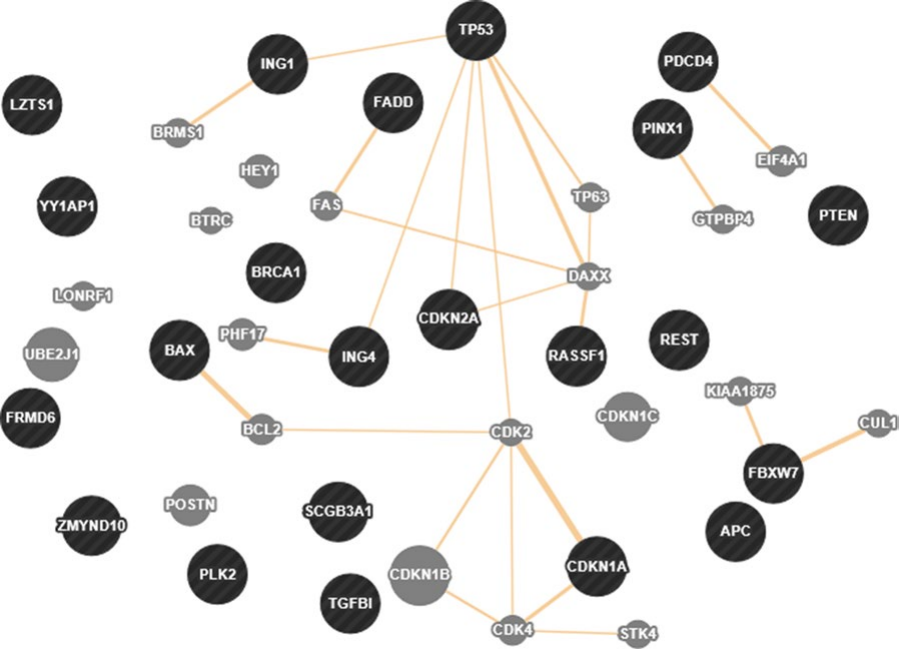
The predicted network of the 22 TSGs based on GeneMANIA. Predicted: predicted functional relationships between genes, often protein interaction

**Fig. 4:**
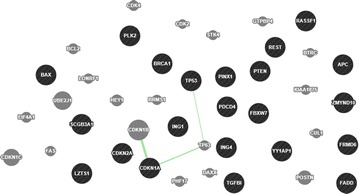
The genetic interactions network of the 22 TSGs based on GeneMANIA. Genetic interaction: Two genes are functionally associated if the effects of perturbing one gene were found to be modified by perturbations to a second gene

**Fig. 5:**
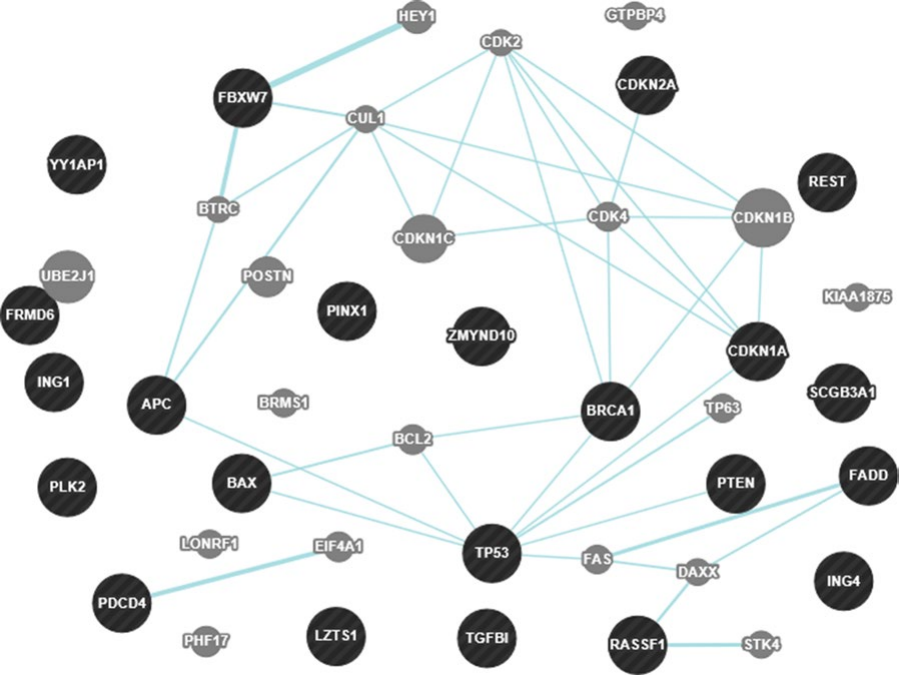
The pathway network of the 22 TSGs based on GeneMANIA. Pathway: Two gene products are linked if they participate in the same reaction within a pathway

**Fig. 6:**
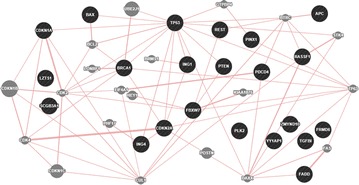
The physical interaction network of the 22 TSGs based on GeneMANIA. Physical Interaction: two gene products are linked if they were found to interact in a protein–protein interaction study

**Fig. 7:**
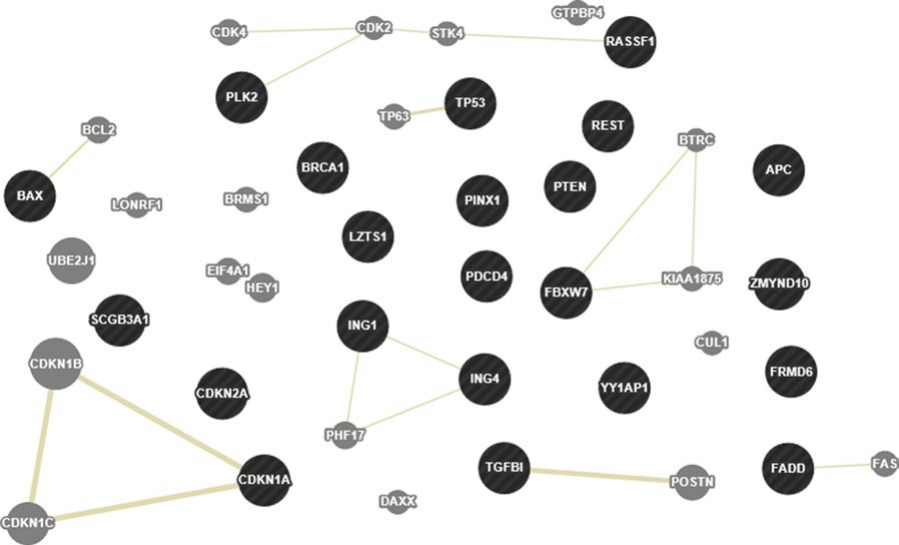
The shared protein domain network of the 22 TSGs based on GeneMANIA. Shared protein domains: Two gene products are linked if they have the same protein domain

Bioinformatics analysis has been widely used in nature and life sciences, and it is a feasible and valuable method for gene/protein function prediction. Numerous networks of molecular interactions have made it possible to study gene/protein function using online databases [80]. GeneMANIA is a web-based interface for prediction of gene/protein function on the basis of multiple networks derived from different proteomics and genomic data, and it is fast enough to predict gene/protein function with a significant accuracy rate [81]. The protein interactions of the 22 genes were analyzed using GeneMANIA. The co-localization, co-expression, pathway, shared protein domains and genetic, physical and predictive interactions of the 22 TSGs were shown in Figs. 1, 2, 3, 4, 5, 6, 7 (Genes/proteins are depicted as colored circles and experimentally detected relationships between genes/proteins as connecting lines. Black circles are the 22 TSGs, gray circles are other genes/protein related to the 22 TSGs). In detail, *BRCA1* has similar expression level with *CDKN2A*, participates in the same pathway with *TP53*, and has physical interactions with *CDKN2A* and *TP53*. *TP53* has similar expression level with *FADD*, *PDCD4*, *CDKN1A* and *Bax*, participates in the same pathway with *BRCA1*, *APC*, *CDKN1A*, *PTEN* and Bax, and has physical interactions with *BRCA1*, *ING1*, *CDKN1A,ING4*, *Bax* and *CDKN2A*. *PTEN* has similar expression level with *PDCD4*, *FBW7,TP53*, *PLK2*, *APC* and *TGFBI*, participates in the same pathway with *TP53*, and has physical interactions with *TP53*, *FBW7*. *APC* has co-localization with *FBW7* and the similar expression level with *FBW7,hEx*, *PTEN* and *REST*, and participates in the same pathway with *TP53*. *CDKN1A* has the similar expression level with *TGFBI*, *ING1*, *FADD*, *TP53*, *CDKN2A* and *PLK2*, and the same protein domain and physical interaction with *TP53*. *CDKN2A* has the similar expression level with *CDKN1A*, *CDKN2A*, and physical interactions with *ING1*, *BRCA1*, *TP53*, *ING4*. *hEx* has the similar expression level with *FBW7*, *ING1,RASSF1*, *PLK2*, *REST*, *PDCD4* and *TGFBI*. *RASSF1* has the similar expression level with *BLU*, *ING4* and *TGFBI*. *YAP* has the similar expression level with *TGFBI*, *PinX1*, *ING1*, *ING4* and *FADD*. *ING4* has the similar expression level with *YAP*, *RASSF1*, *FADD*, *PDCD4*, *Bax* and *PLK2*, the same protein domain with ING1 and physical interactions with *TP53* and *CDKN2A*. HIN-1 has the similar expression level with *FADD*, *REST*, *LZTS1* and *BLU*. *PLK2* has the same protein location with *TGFBI*, and the similar expression level with *TGFBI*, *PTEN*, *LZTS1*, *ING4*, *CDKN1A*, *ING1*, *hEx* and *FBW7*. *LZTS1* has the similar expression level with *PLK2*, *FBW7* and *HIN*-*1*. *FBW7* has the same protein location with *APC*, interacts with *REST* and *PTEN*, and has the similar expression level with *PLK2*, *APC*, *TGFBI*, *LZTS1*, *PDCD4*, *CDKN1A*, *PTEN*, *REST* and *Bax*. *BLU* has the similar expression level with *RASSF1*, *PDCD4*, *HIN*-*1* and *REST*. *TGFBI* has the similar expression level with *FBW7*, *hEx*, *Bax*, *PTEN*, *CDKN1A*, *YAP* and *RASSF1*. *REST* has the similar expression with *BLU*, *APC*, *PinX1*, *FBW7*, *FADD* and *HIN*-*1*. *FADD* has the similar expression of *ING1*, *PinX1*, *CDKN1A*, *ING4*, *HIN*-*1*, *REST*, *YAP*, *Bax* and *TP53*. *PDCD4* has the similar expression with *FBW7*, *TP53*, *ING4*, *BLU* and *PTEN*. *Bax* has the similar expression with *ING4*, *PinX1*, *TP53*, *CDKN1A*, *ING1*, *FBW7*, *FADD* and *TGFBI*, and participates in the same pathway with *TP53*. *PinX1* has the similar expression level with *YAP*, *REST*, *Bax* and *FADD*. *ING1* shares the same protein domain with *ING4* and interacts with *TP53* and *CDKN2A*. The cross-interaction of the 22 TSGs demonstrates that these genes may contribute to PTX resistance as a group.

### Annotated molecular functions of the 22 TSGs according to the protein interaction network

The molecular function of the 22 TSGs may be predicted by using GeneMANIA network. By analysis, there were four summarized functions to be possibly close to chemotherapy resistance (Table 2). The cell cycle-related function covered 12 of the 22 TSGs and additional 10 genes. The cell apoptosis-related function covered eight TSGs and additional seven genes. The protein ubiquitination-related function covered six TSGs and additional five genes. The cell growth-related function covered seven TSGs and additional four genes. Since the cell cycle-related function was annotated with the highest false discovery rate (FDR), the regulation function of TSGs on cell cycle was considered more closely related to chemotherapy resistance than other three pathway functions.

**Table 2:** Summarized molecular functions of the 22 TSGs according to the protein interaction network

Related function	False discovery rate	Number of the 22 TSGs	Other genes
Cell cycle	7.14e^−12^ ~ 4.48e^−2^	*PLK2*, *FBW7*, *TP53*, *PTEN*, *APC*, *BRCA1*, *CDKN1A*, *CDKN2A*, *ING4*, *RASSF1*, *PDCD4*, *FADD*	*CUL1*, *CDK2*, *CDK4*, *CDKN1C*, *CDKN1B*, *GTPBP4*, *TP63*, *PHF17*, *BRMS1*, *STK4*
Apoptosis	1.17e^−9^ ~ 1.16e^−2^	*APC*, *REST*, *CDKN2A*, *FADD*, *ING4*, *TP53*, *Bax*, *BRCA1*	*BRMS1*, *TP63*, *FAS*, *STK4*, *CDKN1B*, *BCL2*, *CUL1*
Protein ubiquitination	4.97e^−9^ ~ 4.39e^−2^	*BRCA1*, *FBW7, TP53*, *CDKN2A*, *RASSF1*, *PTEN*	*BTRC*, *CUL1*, *DAXX*, *GTPBP4*, *CDK2*
Cell growth	1.29e^−8^ ~ 4.25e^−5^	*PTEN*, *ING4*, *TP53*, *CDKN1A*, *CDKN2A*, *ING1*, *HIN*-*1*	*CDKN1B*, *PHF17*, *BCL*-*2*, *BRMS1*

### Other related genes in the network

In the network, 20 other related genes/proteins that mediate the relationship of the 22 TSGs. They have interactions with the 22 candidate genes, and participate in the similar biological process which the 22 genes are involved (Table 2), which indicates that they may be related to PTX or other drug resistance in cancers. Some of them had been studied in drug resistance. For example, tumor protein 63 (*TP63*), a *TP53* family protein, which expressed a variety of isoforms. *DeltaNp63alpha* belonged to the members of the N-terminally truncated (DeltaN) p63 subfamily, it can trigger anti-apoptotic related pathway result to chemo-resistance in hepatocellular carcinoma [82]. Ovarian cancer cell line with acquired resistance to carboplatin revealed low levels of the gene cyclin-dependent kinase inhibitor 1C (*CDKN1C*), and demethylation agent can reverse the silencing of *CDKN1C* and increased the apoptotic response to carboplatin [83]. *Bcl*-*2*, is specifically considered as an important anti-apoptotic protein and classified as an oncogene, the expression of *Bcl*-*2* can affect PTX induced apoptosis [84]. The Bcl-2/Bax ratio has shown a significant increase in PTX-resistance cancer cells [56, 57]. Hairy/enhancer-of-split related with YRPW motif protein 1 (*HEY1*), cyclin-dependent kinase inhibitor 1B (*CDKN1B*) and fas cell surface death receptor (*Fas*) acted as a part of signaling pathways involved in drug resistance [85]. Death-associated protein 6 (*DAXX*) has been shown to regulate PTX-sensitivity in tumor [86]. *CDK4* and *CDK2* are not only involved in the formation of tumor resistance but also the inhibitors of cyclin-dependent kinase which has been the anti-cancer agent used in clinic [87].

## Conclusion

It is well known that TSGs play an important role in cell cycle, angiogenesis and signal transduction, and currently TSGs are also considered to participate in the formation of chemo-resistance. In this review, we reported an overview of the 22 TSGs associated with PTX resistance in cancer. The status and ways of TSGs to regulate PTX resistance in several types of cancer were integrated in Table 1. Using GeneMANIA, the interaction analysis of TSGs was performed and it was shown that cell cycle might be the main manner for the participation of TSGs in PTX resistance in human cancers, and the 22 TSGs had a direct or indirect relationship with each other and could contribute to PTX resistance as a group.

Therefore, profiling the TSGs status of individual tumors, such as mRNA levels and protein levels, is critical in guiding the optimization of personalized medicines to better sensitize the individual patient to specific drugs. Understanding the mechanistic basis and identification of robust biomarkers could also predict optimal use of chemotherapy in patients. We anticipate that in the future such approaches will benefit clinical development of anti-cancer therapeutics directly or indirectly targeting TSGs.

## Abbreviations

APC: adenomatous polyposis coli
AKT: protein kinase B
BRCA1: breast cancer 1
BLU: zinc finger MYND domain-containing protein 10
BCL-2: B cell CLL/lymphoma 2
BTRC: beta-transducin repeat containing E3 ubiquitin protein ligase
BRMS1: breast cancer metastasis suppressor 1
CDK1: cyclin-dependent kinase 1
CDK2: cyclin-dependent kinase 2
CDK4: cyclin-dependent kinase 4
CDK6: cyclin-dependent kinase 6
CDKN2A: cyclin-dependent kinase inhibitor 2A
CDKN1A: cyclin-dependent kinase inhibitor 1A
CDKN1C: cyclin-dependent kinase inhibitor 1C
CDKN1B: cyclin-dependent kinase inhibitor 1B
CKIs: cyclin-dependent kinase inhibitors
CUL1: cullin 1
DAXX: death-associated protein 6
ESCC: esophageal squamous cell carcinoma
FDR: false discovery rate
FBW7: f-box and WD repeat domain containing 7
FADD: phosphorylation of the Fas-associated death domain
FAS: fas cell surface death receptor
GTPBP4: GTP binding protein 4
hEx: human homolog of drosophila expanded
HNSCC: human brain and neck squamous cell carcinoma
HIN-1: high in normal-1
HEY1: hairy/enhancer-of-split related with YRPW motif protein 1
ING1: inhibitor of growth family member 1
ING4: inhibitor of growth 4
iASPP: inhibitor of apoptosis-stimulating protein of p53
JNK: c-Jun N-terminal kinase
LZTS1: leucine zipper putative tumor suppressor 1
mir-22: microRNA 22
mir-135a: microRNA 135a
MDR: multiple drug resistance
MMTV-PyMT: mouse mammary tumor virus promoter-polyoma middle T-antigen
MAPK: mitogen-activated protein kinase
NSCLC: non-small-cell carcinoma
PTX: paclitaxel
PTEN: phosphatase and tensin homolog
PinX1: the telomere/telomerase binding factor
PDCD4: programmed cell death protein 4
PI3 k: phosphatidylinositol 3-kinase
PLK2: polo-like kinase 2
PIP3: phosphatidylinositol(3,4,5)-trisphosphate
PUMA: p53 upregulated modulator of apoptosis
PHF17: PHD finger protein 17
Rb: retinoblastoma
REST: RE1-silencing transcription factor
RASSF1: ras association domain-containing protein 1
SAPK: stress-activated protein kinase
siRNA: small interfering RNA
STK4: serine/threonine kinase 4
TP53: tumor protein 53
TP63: tumor protein 63
TGFBI: transforming growth factor-β-induced
TSGs: tumor suppressor genes
TAZ: transcriptional co-activator with PDZ-binding motif
TUBB3: tubulin beta 3 class III
YAP: yes-associated protein
